# Transplantation of Hematopoietic Stem Cells Promotes Functional Improvement Associated with NT-3-MEK-1 Activation in Spinal Cord-Transected Rats

**DOI:** 10.3389/fncel.2017.00213

**Published:** 2017-07-19

**Authors:** Liu-Lin Xiong, Fei Liu, Shi-Kang Deng, Jia Liu, Qi-Qin Dan, Piao Zhang, Yu Zou, Qing-Jie Xia, Ting-Hua Wang

**Affiliations:** ^1^Institute of Neurological Disease, Department of Anesthesiology, Translational Neuroscience Center, West China Hospital, Sichuan University Chengdu, China; ^2^Institute of Neuroscience, Kunming Medical University Kunming, China

**Keywords:** hematopoietic stem cells, spinal cord transection, cell transplantation, neurological behavior, neurotrophin 3, MEK-1

## Abstract

Transected spinal cord injury (SCT) is a devastating clinical disease that strongly affects a patient’s daily life and remains a great challenge for clinicians. Stem-cell therapy has been proposed as a potential therapeutic modality for SCT. To investigate the effects of hematopoietic stem cells (HSCs) on the recovery of structure and function in SCT rats and to explore the mechanisms associated with recovery, 57 adult Sprague-Dawley rats were randomly divided into sham (*n* = 15), SCT (*n* = 24), and HSC transplantation groups (*n* = 15). HSCs (passage 3) labeled by Hoechst 33342, were transplanted intraspinally into the rostral, scar and caudal sites of the transected lesion at 14 days post-operation. Both *in vitro* and *in vivo*, HSCs exhibited a capacity for cell proliferation and differentiation. Following HSC transplantation, the animals’ Basso, Beattie, and Bresnahan (BBB). locomotion scale scores increased significantly between weeks 4 and 24 post-SCT, which corresponded to an increased number of 5-hydroxytryptamine (5-HT) fibers and oligodendrocytes. The amount of astrogliosis indicated by immunohistochemical staining, was markedly decreased. Moreover, the decreased expression of neurotrophin- 3 (NT-3) and mitogen-activated protein kinase kinase-1 (MEK-1) after SCT was effectively restored by HSC transplantation. The data from the current study indicate that intraspinally administered HSCs in the chronic phase of SCT results in an improvement in neurological function. Further, the results indicate that intraspinally administered HSCs benefit the underlying mechanisms involved in the enhancement of 5-HT-positive fibers and oligogenesis, the suppression of excessive astrogliosis and the upregulation of NT3-regulated MEK-1 activation in the spinal cord. These crucial findings reveal not only the mechanism of cell therapy, but may also contribute to a novel therapeutic target for the treatment of spinal cord injury (SCI).

## Introduction

Spinal cord injury (SCI) is a devastating clinical disorder that usually results severe damage to sensory, motor and autonomic functions distal to the level of the trauma (Hirano et al., 2012; Kamei et al., 2012; Vawda and Fehlings, 2013; Dasari et al., 2014). Current treatments consist of decompressing and stabilizing the injury, preventing secondary complications and rehabilitating function (Bavaria et al., 2007; Tederko et al., 2009), but the efficacy of these treatments is limited. As a result, most SCI patients suffer from substantial neurological dysfunction and lifelong disability (Coutts and Keirstead, 2008; Bracken, 2012). Therefore, it is important to study the underlying mechanisms of and find new therapies for SCI.

Stem cell transplantation, an effective method of biotherapy, has been used to improve nervous system disorders (Mothe and Tator, 2012; Vawda and Fehlings, 2013). Hematopoietic stem cells (HSCs; Aggarwal et al., 2012), a type of multipotent stem cell that can give rise to all types of blood cells and lymphoid lineages, have been used to treat various hematological disorders such as severe combined immunodeficiency, congenital neutropenia (Tyndall et al., 1999; Burt et al., 2008), and malignancies (Mendez et al., 2005). Recently, HSC transplantation (Huang et al., 2010), has also been used as a valuable therapeutic intervention for stroke, traumatic brain injury and multiple sclerosis (MS; Burt et al., 1995), and to study the underlying mechanisms involved in transdifferentiation, neuroprotection through trophic support and cell fusion (Haas et al., 2005), as well as the replacement of lost or damaged cell populations (Nishio et al., 2006; Schwarting et al., 2008; Xu and Onifer, 2009; Mothe and Tator, 2012; Boulland et al., 2013; Vawda and Fehlings, 2013; Nicaise et al., 2015; Singh, 2015). In addition, several previous studies have indicated that transplantation of HSCs from bone marrow or human umbilical cord blood (HUCB) could effectively promote the repair of the spinal cord in animals 1 week post-injury (Koshizuka et al., 2004; Cabanes et al., 2007; Dasari et al., 2008; Deda et al., 2008). Moreover, HSCs have been used in the treatment of SCI patients in clinic, and have shown beneficial effects in reducing deterioration after SCI (Deda et al., 2008; Bryukhovetskiy and Bryukhovetskiy, 2015; Thakkar et al., 2016). Bryukhovetskiy and Bryukhovetskiy (2015) demonstrated the safety and effectiveness of HSCs transplantation in 202 cases of SCI, showing that the administration of HSC can effectively improve the quality of life for SCI patients. However, the concrete mechanisms underlying the effect of HSC transplantation for both morphological remodeling and molecular alternation remain to be understood. Previously, it has been shown that vascular endothelial growth factor (VEGF), and stromal cell-derived factor-1 (SDF1), and its receptor, Cxc chemokine receptor 4 (CXCR4) were involved in the effects caused by HSC transplantation (Deda et al., 2008). However, crucial evidence linking HSCs to improvement in neural behavior in terms of both morphology and at the molecular level is too limited.

Neurotrophic factors (NTFs) play a vital role in nerve regeneration, neovascularization and growth and differentiation of neurons and non-neuronal cells (Lambert et al., 2004; Feng et al., 2005). Persistent delivery of NTFs is crucial for creating a microenvironment for cell survival and nerve regeneration in SCI (Li et al., 2016). Accumulating evidence has demonstrated a crucial role for NTFs in the treatment of neurological diseases after cell transplantation (Isele et al., 2007; Müller et al., 2009; Abbaszadeh et al., 2015), but the role of Neurotrophin-3 (NT-3), an important member of the NTF family, in SCI following with HSC transplantation needs to be explored.

As previous reports have revealed that NT-3 can promote proprioceptive axon regeneration in the injured spinal cord (Li et al., 2016; Liu et al., 2016; Keefe et al., 2017) and that the mitogen-activated protein kinase kinase/extracellular signal-regulated kinase/cAMP-response element binding protein (MEK/ERK/CREB) signaling pathway is crucial for neuroprotection (Lebesgue et al., 2009), the current study was designed to evaluate the role of HSC transplantation in the transected spinal cord, and to explore associated mechanisms involving NT-3-MEK signaling so as to provide translational evidence for the usage of HSCs in future clinical trials. The results revealed that HSC transplantation can improve the neurological function of transected spinal cord injury (SCT) rats by enhancing 5-hydroxytryptamine (5-HT) positive fibers and oligogenesis, suppressing excessive astrogliosis, and upregulating NT3-regulated MEK-1 activation in the spinal cord. These findings reveal the mechanism of cell therapy and contribute to a novel therapeutic target for the treatment of SCI.

## Materials and Methods

### Animal Protocol

Fifty-seven adult male Sprague-Dawley (SD) rats, weighing 220 ± 20 g, were provided by the Center of Experimental Animals, Kunming Medical University. Animals were provided access to pellet chow and water *ad libitum* and were housed in individual cages in a temperature (21–25°C) and humidity (45%–50%) controlled room with a 12-h light/dark cycle. In addition, following SCT, rats were placed in warm condition to keep body temperatures and the bladders were manually massaged three times a day to enhance their function.This study was carried out in accordance with the recommendations of guidelines for laboratory animal care and safety from the United States National Institutes of Health. The protocol was approved by the guidelines of the Institutional Medical Experimental Animal Care Committee of Sichuan University, West China Hospital, China.

The rats were randomly divided into three groups. The sham group (*n* = 15) received no transection or transplantation; the SCT group (*n* = 15) had SCT performed and treated with Dulbecco’s Modified Eagle Medium: nutrient mixture F-12 (DMEM/F12; Hyclone, Logan, UT, USA); the transplantation group (*n* = 24) were subjected to SCT and then injected with an HSC suspension. The assignment of cases is shown in Table 1.

**Table 1:** The number of animals in each group.

Group	BBB evaluation (week 1–24 post SCT)	HSCs survival detection (1 m/3 m/6 m post SCT)	qRT-PCR 6 m post SCT	IHC/IF 6 m post SCT
	*n*	*n*	*n*	*n*
SCT (*n* = 15)	15	-	8	7
HSC (*n* = 24)	15	3/3/3	8	7
Sham (*n* = 15)	15	-	8	7

*SCT, transected spinal cord injury with culture medium; HSCs, hematopoietic stem cells; qRT-PCR, quantitative reverse transcription-polymerase chain reaction; IHC, immunohistochemistry; IF, immunofluorescent staining; 1 m/3 m/6 m, 1 month/3 months/6 months*.

### Transected Spinal Cord Injury Model

The SCT procedure was established as previously described (Liu et al., 2014). Briefly, rats were anesthetized intraperitoneally with 2% sodium pentobarbital sodium at a dose of 30 mg/kg. A laminectomy of T9-11 was subsequently performed, and the dura was opened with a surgical blade to expose a length of spinal cord about approximately 1.5 cm long. Complete transection of the T10 spinal cord was performed and the intervening tissue was removed. The completeness of the transection was assured by lifting the cut ends with small forceps. Sham animals underwent the same surgical procedures except for the T10 transection. After transecting the spinal cord, the surgery incision was sutured. The animals’ hind limbs became motionless after their surgery, remaining in a condition of paraplegia (Gruner, 1992). Following the procedure, 5% cefotaxime sodium salt (diluted 10× with normal saline) was injected intraperitoneally (0.5 μl per rat) once per day until the post-operative day 7. The bladders were manually massaged three times a day until recovery of micturition reflex.

### Cell Isolation and Identification

Three SD rats were sacrificed routinely after being anesthetized as described above, and the femurs were removed. Bone marrow was obtained from femoral bones as previously reported (Sasaki et al., 2001; Koda et al., 2005). Briefly, the epiphyses of the femurs were removed, and the femurs were dissected down the midline. The marrow was then extruded using a syringe filled with DMEM/F12 containing 10% fetal bovine serum (FBS, Gibco, Carlsbad, CA, USA) to obtain the amount of marrow from the head of the femur. Afterward, the bone marrow was beaten into a single cell suspension with 5 ml DMEM/F12 containing 10% FBS and 10,000 U/L penicillin and 10 mg/L streptomycin. After centrifuging (1000 rpm, 5 min) and re-suspending, cells were plated in a 75 cm^2^ culture flask at a density of 1 × 10^6^ cells/ml and incubated for 12 h (37°C, 95% humidity, 5% CO_2_). The suspended cells were then collected in a flask and transferred to another culture flask for further incubating. Culture media was changed twice per week. When the cells grew to a density of between 4 and 5 × 10^6^ cells/cm^2^, they were passaged. Only suspended cells were collected for 2–3 weeks following cultivation due to a lack of additional adherent cells any more. Thus, following the third passage, we were left with pure suspended HSCs. After culturing for 3 days, the HSCs were identified by CD34 enzyme histo-cytochemical staining.

### Cell Marking

HSCs were labeled by Hoechst 33342 2 h prior to transplantation. Briefly, a fluorescent dye was added after replacing the media, then the cells were washed with phosphate-buffered saline (PBS; no Ca^2+^ or Mg^2+^) after being incubated at 37°C with 5% CO_2_ for 2 h. After incubation, DMEM/F12 was added and the cells were counted and concentrated at a final concentration of 2.0 × 10^4^/μl.

### Cell Transplantation

Each rat was anesthetized and placed in a stereotaxic frame. Six sites around the rostral, scar and caudal area of the transected spinal cord were injected using the following coordinates: two sites 5 mm rostral and two sites 5 mm caudal to the injured site, two sites located in the injured site. At a rate of 600 nl/min, a total of 6 μl of the cell suspension was injected into the spinal cord through a glass micropipette positioned at 60 degrees, with 1 μl of the cell suspension (2.0 × 10^4^/μl) injected at each site. After injection, the glass pipette remained in its position for 5 min before being slowly retracted. The control group underwent the same procedure using DMEM/F12. Then, the rat was removed from the device, and the incision was sutured. For suppressing the immunoreaction, cyclosporin-A (10 mg/kg per day) was intraperitoneally used from the third day before transplantation, and kept till the animals were sacrificed.

### Behavioral Assessment

From week 1 to 24 post-SCT, Basso, Beattie and Bresnahan. (BBB) locomotor rating scale values were recorded in an open enclosure (99 cm in diameter, 23 cm deep) with scores graded from 0 points (absence of any hind limb movement) to 21 points (normal mobility Basso et al., 1995). In order to avoid motionlessness upon introduction to a new environment, subjects were acclimated to the observation fields for 3 days prior to surgery for 5 min per day. During testing, each subject was placed in the open field and observed for 4 min (He et al., 2016). The final score was the average of three individual researchers, who were blinded to the experimental treatment.

### Tissue Harvest

Twenty-four weeks after SCT, rats were anesthetized with 3.6% chloral hydrate (1 ml/100 g, intraperitoneal injection) and transcardially perfused with heparinized physiological saline followed by 4% paraformaldehyde in 0.1 M ice-cold phosphate buffer, pH 7.4. Immediately after perfusion, a length of spinal cord extending from 10 mm rostral to 10 mm caudal to the injured site was collected. Samples for quantitative real-time polymerase chain reaction (qRT-PCR) were harvested and stored in 1.5 ml RNase-free Eppendorf tubes at −80°C. Spinal cords for immunohistochemical staining were post-fixed for 5 h at 4°C. The tissues were stored in 30% sucrose in 0.1 M phosphate buffer, pH 7.4, for 72 h at 4°C. Then the rostral, scar and caudal segments of transected spinal cord from the different groups were embedded in the same paraffin block and sectioned at 5 μm thickness. After routinely deparaffinized and rehydrated, immunohistochemistry was performed on the slices of spinal cord tissue. Some slices were placed under a fluorescent microscope for direct observation of the state of the transplanted cells.

### Immunofluorescence Observation of HSC Differentiation

To observe the differentiation of HSCs into neurons and astrocytes *in vitro* and *in vivo*, immunofluorescence staining of Tuj1 and aldehyde dehydrogenase 1 family, member L1 (ALDH1L1) was performed, respectively. In brief, the primary antibodies directed against Tuj1 (1:100, Rabbit, ABclonal, College Park, MD, USA) and ALDH1L1 (1:100, Rabbit, ABclonal Biotech), as well as the secondary antibody Dylight 594 (1:100, goat anti-rabbit, Abbkine) were applied progressively as previously described (Liu et al., 2014). A negative control was performed by using PBS in place of the primary antibodies. Slides were then viewed and photographed using a fluorescence microscope (Leica, Germany).

### Enzyme Histocytochemical Staining

Immunohistochemistry was used to determine the purity of cultured HSCs (CD34 immunohistochemical staining) and observe the alternations in the oligodendrocyte precursor cells after HSC transplantation into the spinal cord. Oligodendrocytes, neurons and astrocytes were identified using APC, NeuN and GFAP antibodies, respectively. In addition, 5-HT fibers were also identified. Briefly, slices were washed in 0.01 mol/L PBS repeated three times for 5 min each, then incubated at 37.5°C in 3% hydrogen peroxide for 30 min in the dark to block the action of endogenous peroxidases. This was followed by a 5-min immersion in PBS, repeated three times, then immersion in 5% goat serum at 37.5°C for 30 min. Afterwards, sections were incubated overnight at 4°C in the primary antibody solutions shown in Table 2. A negative control was prepared by using PBS in place of the primary antibodies. Next, sections were washed three times with 0.01 mol/L PBS Tween-20 (PBST-20) and incubated with PV-9000 reagent 1 for 30 min at 37.5°C, followed by PV-9000 reagent 2 for 30 min at 37.5°C. Subsequently, sections were in incubated in the chromogenic agent 3,3′-Diaminobenzidine (DAB) for 3–7 min in the dark. The sections were rinsed with water, then counter-stained with hematoxylin. Following dehydration, sealing took place using a transparent and neutral gum, and then positive staining was visualized under an inverted phase contrast microscope imaging system. The cell numbers and sizes were analyzed using Image-Pro Plus 6.0 software (Media Cybernetics, Silver Spring, MD, USA).

**Table 2:** Antibodies used in the study.

Antibodies	Manufacturer	Source	Reactivity	Dilution
CD34	Sigma	Mouse	Rat	1:100
5-HT	Immunostar	Rabbit	Rat	1:20,000
GFAP	Millipore	Rabbit	Rat	1:50
APC	Calbiochem	Mouse	Rat	1:200
NeuN	Immunostar	Rabbit	Rat	1:500

### Quantitative Real-Time PCR (qRT-PCR)

Twenty-four weeks post-SCT, sections of spinal cord (10-mm in length, containing the injury and the graft) from the HSC, SCT and sham groups were collected and homogenized to determine the level of MEK-1 and NT3 mRNA. Total RNA was isolated with Trizol reagent (Takara Bio Inc., Otsu, Japan) and was reverse transcribed into cDNA. Subsequently, qRT-PCR of cDNA was performed using the following primers (TaKaRa Company (Japan)): NT3 (forward) 5′-GTCCATCTTGTTTTAT GTGAT-3′, (reverse) 5′-GTGCTCTGGAATTTTCCTT-3′; MEK-1 (forward) 5′-GCAATCCGGAACCAGATCAT-3′, (reverse) 5′-CAGGAATTCTTCCAGCTTTCT-3′. β-actin was used as the internal control. PCR conditions were as follows: initial denaturation at 95°C for 2 min, denaturation at 95°C for 15 s and amplification at 53°C for 20 s, followed by extension at 60°C for 30 s for a total of 40 cycles. The threshold cycle (Ct) of each sample was recorded, and data were analyzed by normalization to β-actin values using the 2^−ΔΔCt^ method (Livak and Schmittgen, 2001; Liu et al., 2014).

### Statistical Analysis

All statistical analyses were performed with SPSS19.0 software (IBM Corporation, NY, USA). Data were analyzed by Student’s *t* test between two groups. For multiple group comparison, one-way ANOVA with Tukey’s *post hoc* multiple comparisons was applied. *P* < 0.05 was considered statistically significant. Data are expressed as mean ± standard deviation (SD).

## Results

### Identification and Morphology of HSCs Cultured *In Vitro*

**Figure 1:**
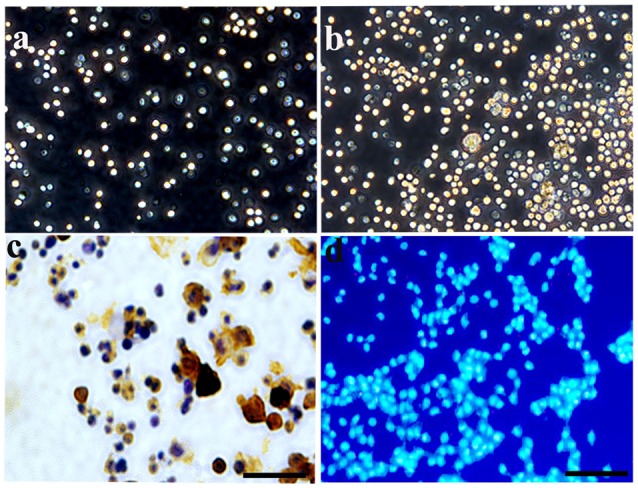
Characterization and identification of hematopoietic stem cells (HSCs) cultured *in vitro*. **(A)** Under a phase contrast microscopy, HSCs exhibited a floating growth status at day 3, and the number increased greatly at day 7 **(B)**. **(C)** Enzyme histo-cytochemical staining at 3 days showed most of the cultured HSCs expressed CD34 demonstrating the purity of the cultured HSC. **(D)** HSCs were labeled by Hoechst 33342 with nucleus staining, showing a blue color. Bar = 50 μm.

At 3 days post-culture, HSCs were round in shape with bright edges and exhibited a floating growth status (Figure 1A). They increased greatly in number 7 days later (Figure 1B). CD34+, a specific marker for HSCs, was used to identify the cultured HSCs at 3 days post-culture. The results showed that CD34+ positive staining was expressed in these cells, and that the positive rate was >90% at 3 days post-culture (Figure 1C), which further confirmed the purity of the HSCs. As cultured HSCs would be transplanted into the host, Hoechst 33342 was also used to label the cultured cells, which showed a blue color with nucleus staining (Figure 1D).

### Differentiation of the Cultured HSCs *In Vitro*

**Figure 2:**
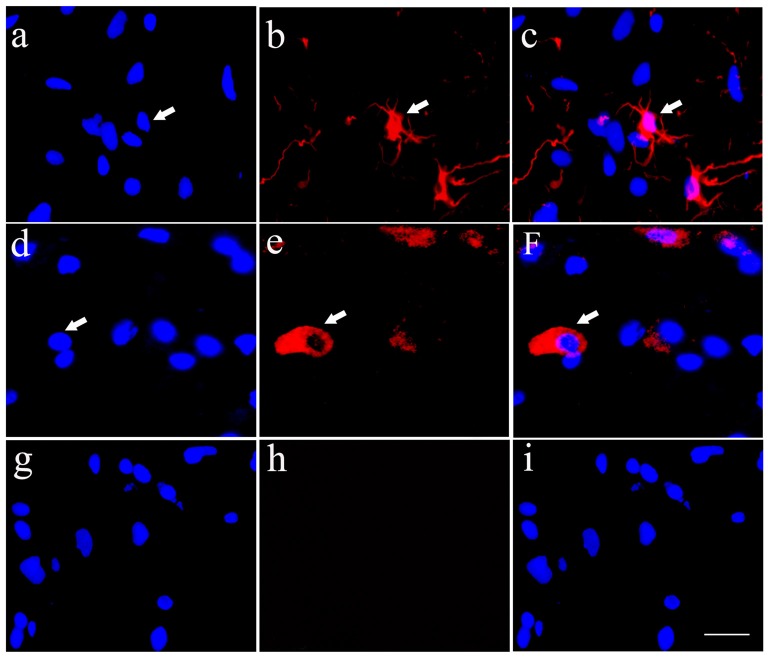
Cultured HSCs differentiate towards neurons and glia cells *in vitro*. Cultured HSCs differentiated toward neurons **(A–C)** and astrocytes** (D–F)**, which were identified by immunofluorecent staining of Tuj1 and aldehyde dehydrogenase 1 family, member L1 (ALDH1L1) antibodies, respectively, while the negative control showed no positive staining **(G–I)**. Bar = 25 μm. White arrows represented the positive cells.

In order to detect the differentiation of HSCs into neurons and astrocytes, an immunofluorecence staining of Tuj1 (Figures 2A–C) and ALDH1L1 (Figures 2D–F), respectively was performed in the cultured HSCs. The results showed that HSCs have the ability to differentiate into neurons and astrocytes. In these experiments, PBS was used as negative control instead of the primary antibody, and resulted in unspecific staining except for a blue nucleus (Figures 2G–I).

### Fate of HSCs in the Host Spinal Cord

**Figure 3:**
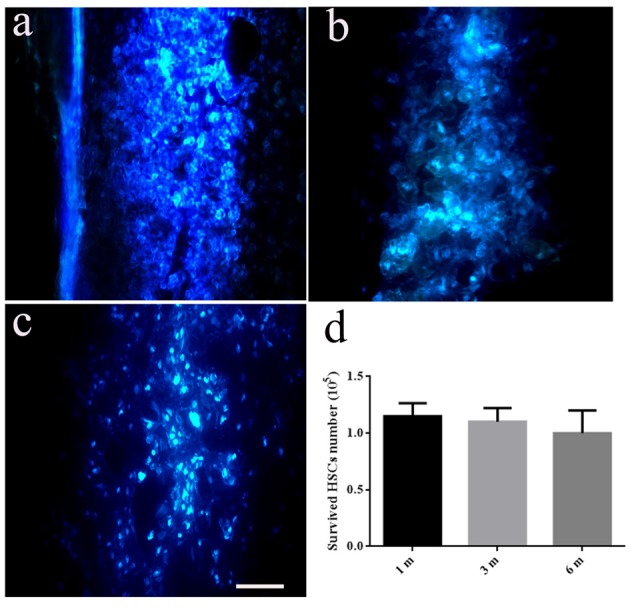
Transplanted HSCs survived and migrated in the host spinal cord. There are no positive HSCs with blue staining marked by Hoechst 33342 in the spinal cord without HSC transplantation. While, HSCs with blue staining in nucleus could be seen at the first month **(A)**, third month **(B)** and sixth month post transected spinal cord injury (SCT) **(C)**, indicating the survival and migration of the transplanted HSCs in the host spinal cord. **(D)** Bar chart for quantitative analysis of the survived HSCs *in vivo* using Image-Pro Plus 6.0 software. Data are presented as the mean ± SD (*n* = 3, one-way ANOVA). Bar = 50 μm. m, month.

There were no Hoechst-positive cells found in the SCT control group. Comparatively, HSCs with blue staining in the nucleus could be seen in HSC transplanted rats at the first month (Figure 3A), third month (Figure 3B) and sixth month (Figure 3C) post-operation. Implanted HSCs also migrated towards the caudal and rostral ends of the cord, and the number of surviving cells was approximately 90% of the total number of transplanted cells at sixth months post-SCT (Figure 3D).

### Differentiation of HSCs Following Transplantation into the Host Spinal Cord

**Figure 4:**
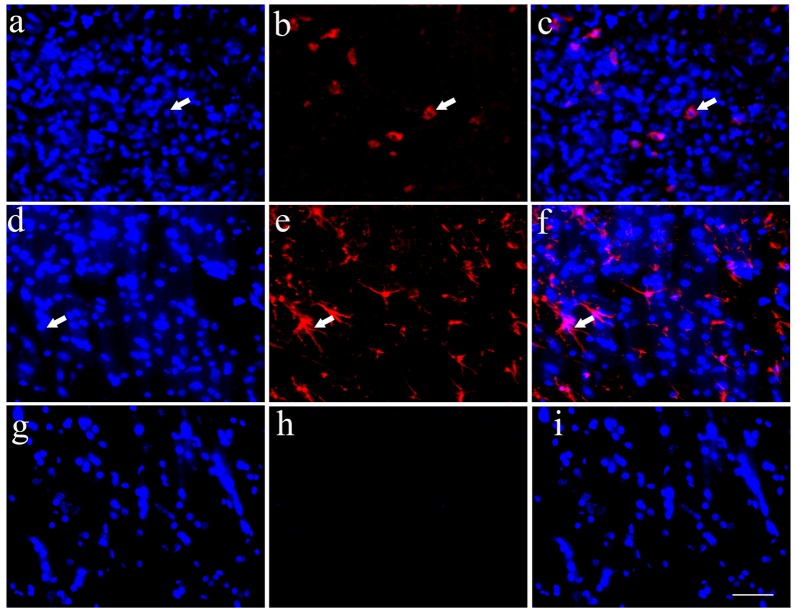
Transplanted HSCs could differentiate into neurons and glia like cells *in vivo*. **(A)** Positive HSCs with blue staining in nucleus marked by Hoechst 33342 in the spinal cord could be seen after HSC transplantation. **(B)** A few of HSCs could differentiate into neurons with Tuj1 positive staining (red). **(C)** The merged picture of Tuj1 and Hoechst 33342, the positive ratio of Tuj1/ Hoechst 33342 is about 3.1%. **(D)** Positive HSCs with Hoechst 33342 blue staining. **(E)** A few of HSCs could differentiate into astrocytes with ALDH1L1 positive staining (red). **(F)** The merged picture of ALDH1L1 and Hoechst 33342, which showed the positive ratio is about 18.3%. **(G)** Hoechst 33342 blue staining in the negative control (no primary antibody). **(H)** There is no positive red staining in the negative control (no primary antibody). **(I)** The merged picture. Bar = 50 μm. White arrows represented the positive cells. The positive ratio of Tuj1 (Tuj1/ Hoechst 33342) and ALDH1L1 (ALDH1L1/Hoechst 33342) was quantified using Image-Pro Plus 6.0 software (*n* = 7).

Implanted HSCs with blue staining labeled by Hoechst 33342 could be found in the host spinal cord, which confirmed the survival of HSCs (Figures 4A,D,G). Simultaneously, some HSCs exhibited positive Tuj1 staining (Figures 4B,C) or ALDH1L1 staining (Figures 4E,F). Quantitative analysis showed that 3.1% of the HSCs were positive for Tuj1 and 18.3% were positive for ALDH1L1. These data demonstrate that the transplanted HSCs can develop into astrocytes *in vivo*, with fewer neuronal differentiations. The negative control showed no positive staining (Figures 4G–I).

### Behavioral Assessment

**Figure 5:**
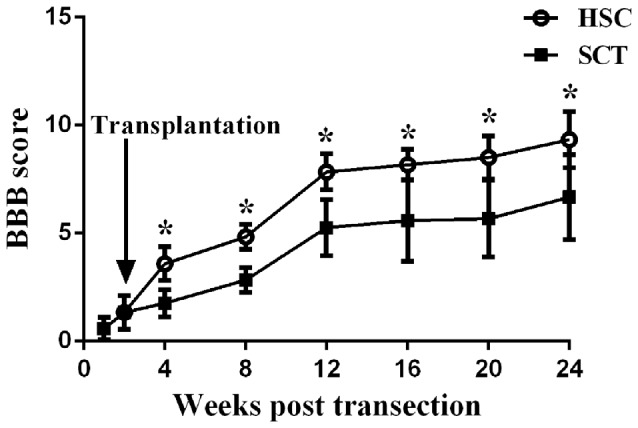
HSCs transplantation improved the neural behavior in rats subjected to SCT. BBB score of the sham group was 21. There was a significant decrease of BBB score at week 1–24 after operation in SCT and HSC group (*n* = 15/group). HSC transplantation group displayed a significantly improved scores compared with SCT group beginning at week 2–22 after transplantation. Three independent experiments were performed and the data are presented as the mean ± SD (*n* = 15). Student’s *t* test was used to analyze the data. *P < 0.05 vs. SCT. Arrow indicates the HSC transplantation time.

BBB scores from week 1 to 24 showed that SCT resulted in a complete locomotor function deficit, and that locomotor function could be partially restored over time. Comparatively, BBB scores in the HSC group were higher than in the SCT group from week 4 to 24 post-SCT. The behavioral evaluation from the BBB scores demonstrated that HSC transplantation dramatically improves the neurological function of SCT rats (*P* < 0.05, Figure 5).

### Morphological Changes in the Spinal Cord following HSC Transplantation

**Figure 6:**
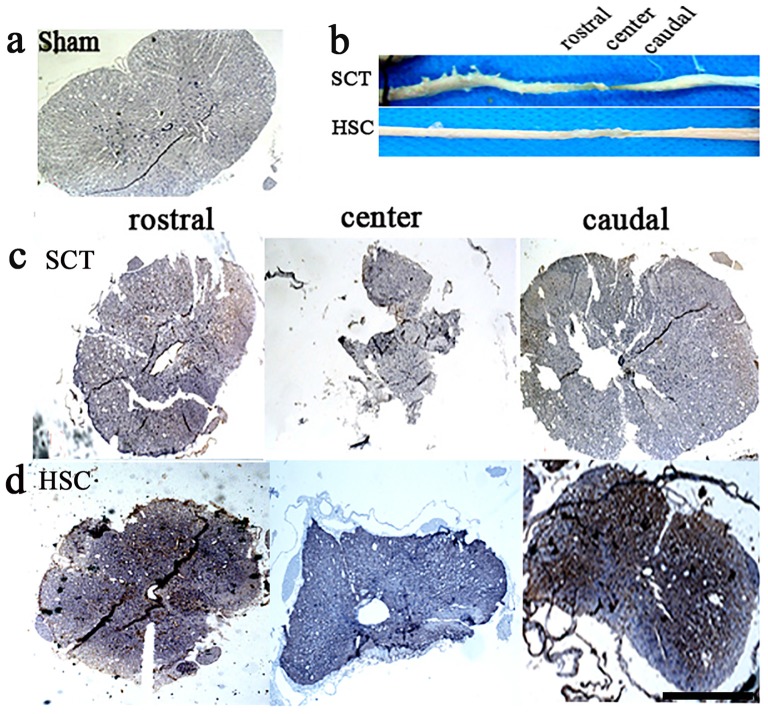
HSC transplantation restored the volume of spared spinal cord. After SCT, compared with the sham one **(A)**, both rostral and caudal spinal cord exhibited a narrow shape, structure was damaged. Moreover, the volume in injured center decreased apparently with the narrowest shape **(B)** upper, **(C)**. Comparatively, HSC transplantation resulted in a significant increase on the volume of spared spinal cord **(B)** down, **(D)**. Bar = 200 μm.

The spinal cord appeared normal in shape, with a plum shape and normal volume, at both the rostral or caudal sides in the sham group at sixth months post-SCT (Figure 6A), while both the rostral and caudal spinal cord in the SCT group exhibited a narrow shape as compared with the sham group. A decreased volume at the center of the injury was most apparent (Figures 6B,C). In contrast, HSC transplantation markedly restored the volume of the spinal cord, and promoted the morphological recovery of the injured spinal cord (Figures 6B,D).

### The Effect of HSC Transplantation on Neural Survival

**Figure 7:**
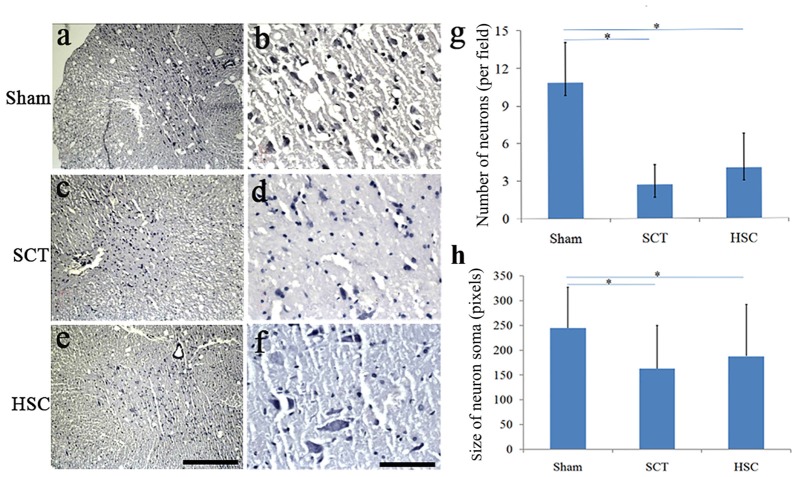
The effect of HSC transplantation on the neural survival. Compared with the sham group **(A,B,G,H)**, the number and size of neurons in ventral horn in the SCT group **(C,D,G,H)** became markedly less. However, quantitative analysis showed no differences on the number and size of neurons between SCT and HSC groups, *P* > 0.05 **(E–H)**. Data are presented as the mean ± SD (*n* = 7). One-way ANOVA was used to analyze the data. *P < 0.05 vs. sham. Bar = 100 μm in **(A,C,E)**; 50 μm in **(B,D,F)**.

Following SCT, the number and size of neurons in layer VIII and IX of the ventral horn became much fewer and smaller than in the sham and HSC group (Figures 7A–D). However, quantitative analysis showed that the cellular number and size in the HSC group was not significantly greater than in the SCT group (*P* > 0.05; Figures 7G,H), which indicated that administration of HSCs did not increase the number and size of neurons in the ventral horn (Figures 7E,F).

### HSC Transplantation Suppressed Astrocyte Proliferation

**Figure 8:**
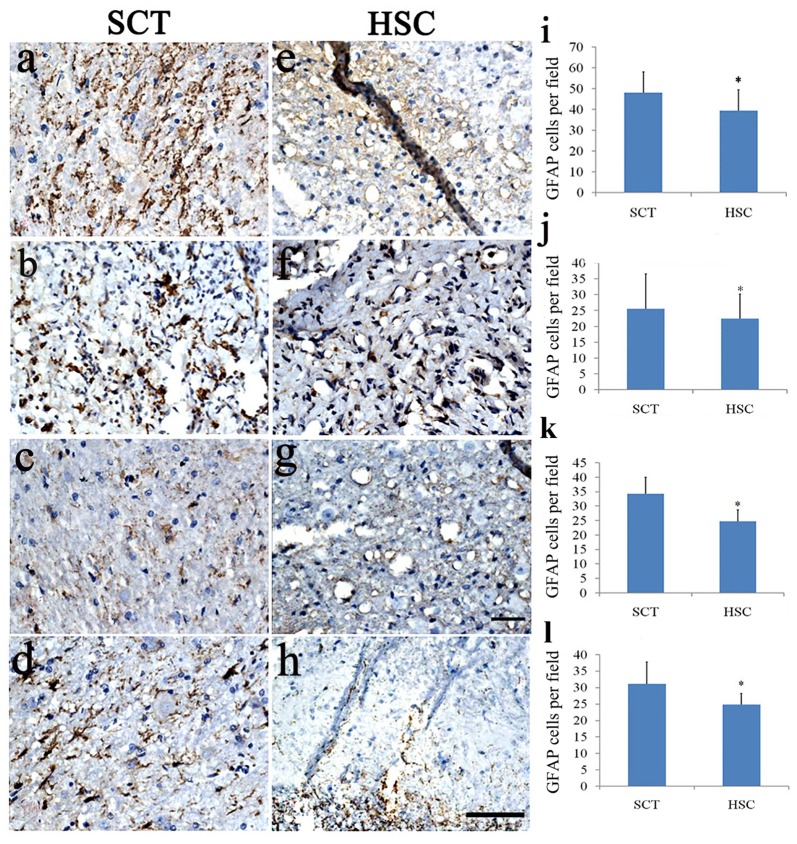
HSC transplantation decreased the number of astrocytes. SCT greatly increased the number of GFAP^+^ cells in the rostral, scar and caudal sites of the transected cord **(A–D)**. While HSCs transplantation effectively impressed the number of astrocytes in these segments **(E–H)**. Histogram showed that the number of astrocytes in HSC transplanted group was lower than that of SCT one **(I–L)**. **(A,E,I)** Scar center; **(B,F,J)** Posterior Funiculus of rostral scar; **(C,G,K)** gray matter of rostral scar; **(D,H,I)** Dorsal horn of caudal scar. Data are presented as the mean ± SD (*n* = 7). Student’s *t* test was used to analyze the data. Data in the sham group were not shown. *P < 0.05 vs. SCT. Bar = 50 μm.

To investigate whether astrocytes were changed after HSC transplantation, double staining of Hoechst 33342 and GFAP was performed in the injured center, and in the rostral and caudal regions 6 months post-SCT. SCT induced a marked increase in the number of GFAP+ cells in the injured center, and rostral and caudal ends of the spinal cord (Figures 8A–D,I–L), compared with the sham group. This finding was confirmed in a previous observation (data not shown). Following HSC transplantation, the number of Hoechst 33342/GFAP-labeled cells effectively decreased in the observed areas (injured center, and rostral and caudal ends of the spinal cord), when compared with the SCT group (*P* < 0.05; Figures 8E–L).

### The Effect of HSC Transplantation on Oligodendrocytes

**Figure 9:**
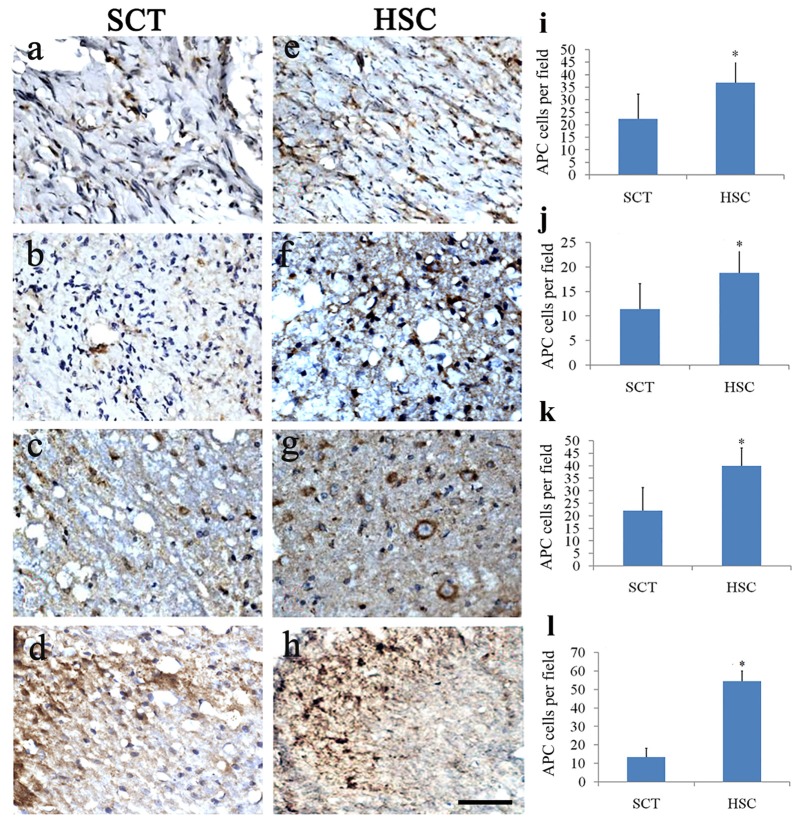
HSC transplantation promoted oligogenesis. Compared with the sham group, the number of APC positive cells was largely decreased in the injured center, rostral and caudal spinal cord near the injured site post SCT **(A–D)**. Comparatively, the number of oligodendrocytes was effectively increased at 24 weeks post-SCT **(E–H)**. Quantitative histogram showed that the number of oligodendrocytes in HSC group was higher than that of the SCT one **(I–L)**. **(A,E,I)** Scar center; **(B,F,J)** Posterior Funiculus of rostral scar; **(C,G,K)** gray matter of rostral scar; **(D,H,I)** Dorsal horn of caudal scar. Data are presented as the mean ± SD (*n* = 7). Student’s *t* test was used to analyze the data. Data in the sham group were not shown. *P < 0.05 vs. SCT. Bar = 50 μm.

Double staining of Hoechst 33342 and APC showed a distinct decrease in the number of olidgoendrocytes in SCT rats compared with the sham group from a previous observation (sham data not shown, Figures 9A–D,I–L). On the contrary, in the HSC group, the number of oligodendrocytes was increased conspicuously when compared with the SCT group (*P* < 0.05; Figures 9E–L). The increase was observed in the scar area as well as neighboring spinal tissue, including the rostral and caudal cords (Figures 9E–L).

### Regeneration of 5-HT Fibers after HSC Transplantation

**Figure 10:**
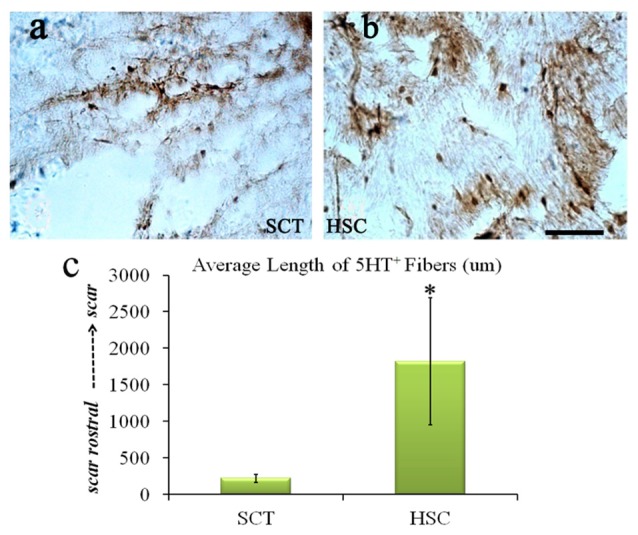
HSC transplantation enhanced the regeneration of 5-HT fibers. After SCT, 5-HT fiber exhibited weak regeneration capacity **(A)**, while HSC transplantation largely increased the number of 5-HT positive fibers **(B)**, the length is near 2 cm **(C)**. Data are presented as the mean ± SD (*n* = 7). Student’s *t* test was used to analyze the data. *P < 0.05 vs. SCT. Bar = 50 μm.

5-HT fibers exhibited a weak regeneration capacity post-SCT compared with the sham group from a previous observation (data not shown). Moreover, 24 weeks post-SCT, the number of 5-HT-positive fibers had increased compared with the SCT group. Descending 5-HT fibers, known for transducing motor signals from the brain to the end effectors, regenerated approximately 2 cm into the scar area (Figure 10).

### Expressional Changes of NT-3 and MEK-1 Signaling in the Spinal Cord Following HSC Transplantation

**Figure 11:**
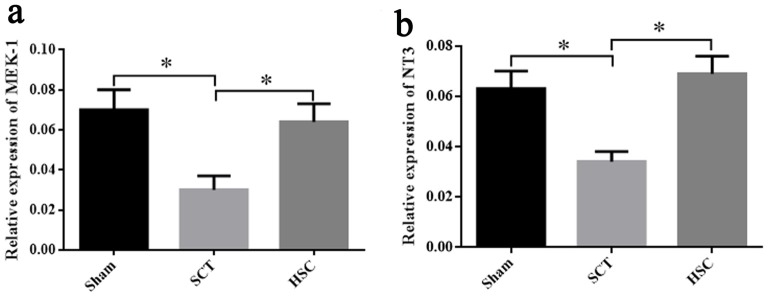
Detection of neurotrophin-3 (NT-3) and mitogenactivated protein kinase kinase-1 (MEK-1) expression after HSC transplantation. The expressional changes of MEK-1 and NT-3 mRNA among the sham, SCT and HSC group at 24 weeks post-SCT were respectively shown in **(A,B)**. *p < 0.05 compared with SCT group. Data are presented as the means ± SEM (*n* = 8). One-way ANOVA was used to analyze the data.

Twenty-four weeks post-SCT, qRT-PCR showed that the mRNA expression of NT3 and MEK-1 in the SCT group was significantly decreased, as compared with the sham group (*p* < 0.05). The levels of NT-3 and MEK-1 mRNA in the HSC group were markedly increased (*p* < 0.05), but were not significantly different from the sham group (*p* > 0.05), when compared with the SCT group. Therefore, increased expression of NT-3 and MEK-1 may be associated with the protection of HSC transplantation (Figures 11A,B).

## Discussion

In the current study, we found that HSC transplantation promotes functional improvement after SCT in rats, and enhances nerve regeneration and oligogenesis while suppressing excessive astrogliosis. Importantly, NT3-regulated MEK-1 activation may be responsible for the success of HSC grafts in SCI rats.

### HSC Transplantation Promotes Functional Improvement Associated with Nerve Regeneration, Oligogenesis and Astrogliosis Inhibition

In this study, we found that HSCs transplanted into SCT rats could survive and migrate around the injured site and ameliorate behavior deficits after SCT. We also found that the decrease in the spinal cord volume of HSC-treated rats was smaller than in SCT rats. It has been shown that the compromised transected tissue and the subsequent cavity formation are characteristics of progressive tissue necrosis following initial primary cell destruction in SCT (Park et al., 2012). Therefore, reduction of the structural injury or cavity volume indicates that the transplanted HSCs have a neuroprotective effect after SCT. Moreover, the results of the present study demonstrate the therapeutic effect of HSCs in chronic SCT rats, which differed from findings of previous studies (Deda et al., 2008; Sasaki et al., 2009). The morphological mechanisms for improving the functional deficits involved in promoting nerve regeneration and oligogenesis, together with astrogliosis inhibition, were shown.

In the previous studies involving animals, reactive astrocytosis was shown to promote the regeneration of severed axons, and that this may have occurred through glial scar-associated extracellular matrix proteins (GrandPré et al., 2000; Bradbury et al., 2002; Kamei et al., 2010, 2012). While the issue of whether reactive astrocytosis is beneficial or detrimental remains controversial, it may play a critical and necessary role in the early stages of destructive CNS processes, but may be harmful in latter stages by contributing to an inhibition of axonal regeneration. Previous studies have also shown that in the repair process of central nervous system damage, an excessive increase in astrocytes promotes the secretion of inhibitory molecules and the formation of fibrotic scarring in the injured spinal cord, which then prevents axonal or nerve regeneration (Frisén et al., 1995; Yu et al., 2000; Shearer and Fawcett, 2001; Fields and Stevens-Graham, 2002). In the current study, we found that the BBB score was improved, astrogliosis was inhibited and 5-HT fibers were increased at 24 weeks post-SCT. These data suggest that the moderate decrease in the number of astrocytes following HSC transplantation may provide an opportunity for nerve regeneration. Our data indirectly show that reactive astrocytosis may be harmful in the latter stages of destructive CNS processes. As our observations lasted 6 months and a decreased number of astrocytes were found, our findings support the hypothesis that HSCs grafts have a protective role in chronic SCI, which is different from previous observations (Deda et al., 2008; Sasaki et al., 2009; Kamei et al., 2010).

Enhanced oligogenesis was also observed after HSC transplantation. It has been suggested that oligogenesis by and survival of endogenous oligodendrocyte progenitor cells (OPCs) can contribute to self-repair after myelin loss (Deda et al., 2008; Park et al., 2012). This finding indicates that oligodendrocytes are important myelin-formation cells responsible for the formation of myelin surrounding axons in the central nervous system. Previous studies have reported that transplanted peripheral blood stem cells mobilized by granulocyte colony-stimulating factor promote hindlimb functional recovery following SCI in mice by suppressing oligodendrocyte apoptosis (Takahashi et al., 2016). Similarly, in the current study, we found that HSC transplantation effectively increased the number of oligodendrocytes and 5-HT fibers *in vivo*. Therefore, the results revealed that HSC transplantation is very important for promotion of functional improvement after SCT by increasing the number of oligodendrocytes and 5-HT fibers. It has been shown that 5-HT fibers promote surviving nerve axons to extend their lateral branches towards damaged axons, and that oligodendrocytes can repair demyelinated CNS neurons and improve the function of spinal cord nerves (Sasaki et al., 2001; Bjugstad et al., 2005; Garbuzova-Davis et al., 2006; Leu et al., 2010), which further supports their role in the functional recovery of the transected spinal cord.

Although HSCs have been proposed as a potential source of neural cells for repairing brain lesions (Sigurjonsson et al., 2005), we found no significant changes in the number or size of neurons in the transected spinal cords treated with HSC transplantation in our research. The degeneration and atrophy of injured neurons post-SCT were difficult to evaluate (Dobkin and Havton, 2004). Moreover, HSC engraftment exhibited the potential to restore injured spinal cord and promote functional recovery, similar to marrow stromal cells in contusion conditions (Koda et al., 2005). The underlying mechanism was involved in promoting the oligogenesis and nerve regeneration from 5-HT fibers, as well as inhibiting excessive astrogliosis.

### Molecular Mechanism Involved in HSC Transplantation

Although the roles of HSC transplantation have been preliminarily determined, the source of HSCs is still an obstacle for their wide application. Thus, the molecular mechanism of HSC transplantation must be explored in order to find alternative treatments. Previous studies have reported that VEGF, SDF1, CXCR4, PI3-K/Akt and MAPK signaling may be involved in protection following HSC transplantation (Isele et al., 2007; Deda et al., 2008). Based on the neuroprotective role of NT-3 and the MEK/ERK/CREB signaling pathway (Isele et al., 2007; Lebesgue et al., 2009; Li et al., 2016; Liu et al., 2016; Keefe et al., 2017), we speculate that HSC transplantation may promote functional improvement after SCT by increasing the expression of NT3 and MEK-1 signaling. Our findings show that SCT induced a significant decline in NT3 and MEK-1 mRNA levels, while HSC transplantation was able to restore this decrease. This indicates that upregulation of NT3 and MEK-1 signaling may be a potential molecular mechanism underlying protection following HSC transplantation. Therefore, activation of NT-3 or MEK-1 signaling may present a new alternative strategy for the treatment of SCI in future clinical practice.

## Conclusion

Our study revealed that HSC transplantation promotes functional improvement in SCT rats and found possible mechanisms that are involved in morphological remodeling and the activation of NT-3 and MEK-1 signaling. These findings may contribute to the understanding of the mechanism of SCI and provide a novel treatment strategy for neural repair following SCI.

## Author Contributions

L-LX, FL, Q-JX and T-HW designed the experiments. L-LX, FL, S-KD, JL, Q-QD and YZ performed the experiments. L-LX, PZ and Q-JX analyzed the data. L-LX, FL and T-HW wrote the manuscript. All authors read and approved the final manuscript.

## Conflict of Interest Statement

The authors declare that the research was conducted in the absence of any commercial or financial relationships that could be construed as a potential conflict of interest.
